# Effects of Genistein on Differentiation and Viability of Human Visceral Adipocytes

**DOI:** 10.3390/nu10080978

**Published:** 2018-07-27

**Authors:** Elena Grossini, Serena Farruggio, Giulia Raina, David Mary, Giacomo Deiro, Sergio Gentilli

**Affiliations:** 1 Laboratory of Physiology, Department of Translational Medicine, University of Eastern Piedmont, Via Solaroli 17, 28100 Novara, Italy; serefar@live.it (S.F.); jiugi@hotmail.it (G.R.); davidmary38@hotmail.com (D.M.); 2 Experimental Surgery, Azienda Ospedaliera Universitaria Maggiore della Carità, Corso Mazzini 36, 28100 Novara, Italy; 3 AGING Project, Department of Translational Medicine, University of Eastern Piedmont, via Solaroli 17, 28100 Novara, Italy; 4 General Surgery Unit, Department of Health Sciences, University of Eastern Piedmont, Via Solaroli 17, 28100 Novara, Italy; giacomo.deiro@gmail.com (G.D.); sergio.gentilli@med.uniupo.it (S.G.)

**Keywords:** adiponectin, browning, cell survival, differentiation, genistein, mitochondria, peroxidation

## Abstract

Obesity can lead to pathological growth of adipocytes by inducing inflammation and oxidative stress. Genistein could be a potential candidate for the treatment of obesity due to its antioxidant properties. Specific kits were used to examine the effects of genistein vs adiponectin on human visceral pre-adipocytes differentiation, cell viability, mitochondrial membrane potential, and oxidative stress in pre-adipocytes and in white/brown adipocytes. Western Blot was performed to examine changes in protein activation/expression. Genistein increased human visceral pre-adipocytes differentiation and browning, and caused a dose-related improvement of cell viability and mitochondrial membrane potential. Similar effects were observed in brown adipocytes and in white adipocytes, although in white cells the increase of cell viability was inversely related to the dose. Moreover, genistein potentiated AMP-activated protein kinase (AMPK)/mitofusin2 activation/expression in pre-adipocytes and white/brown adipocytes and protected them from the effects of hydrogen peroxide. The effects caused by genistein were similar to those of adiponectin. The results obtained showed that genistein increases human visceral pre-adipocytes differentiation and browning, protected against oxidative stress in pre-adipocytes and white/brown adipocytes through mechanisms related to AMPK-signalling and the keeping of mitochondrial function.

## 1. Introduction

Obesity arises from the imbalance between energy intake and energy expenditure, and may lead to a pathological growth of adipocytes [1,2,3,4]. Although the underlying mechanisms remain unclear [5,6], growing evidence suggests that oxidative stress and mitochondrial dysfunction would act as key factor in the pathophysiology of obesity and in the cross talk with its associated complications [2,5,7,8]. Also, brown adipose tissue (BAT), the less represented type of visceral adipose tissue (VAT), could play a role in body fat accumulation [6,9,10]. Hence, BAT cells can generate heat and fatty acid energy substrates thanks to their large numbers of mitochondria in which oxidative phosphorylation is uncoupled from ATP production as a result of a transmembrane proton leak mediated by uncoupling protein 1 (UCP1) [11]. Previous findings have shown that when BAT levels are either increased or decreased, the protection against diet-induced obesity is affected [12].

Extensive studies have focused on the role of nutritional factors in the etiology, prevention, and treatment of obesity. In this context, genistein has emerged as a key agent amongst the various agents examined [4,11,12,13]. Phytoestrogen genistein is one of several known isoflavones of soybeans and it binds directly to estrogen receptors (ERs), thus exerting both estrogenic and anti-estrogenic effects [14]. Recent studies demonstrated that lifelong exposure to dietary isoflavones and phytoestrogens, which is typical of traditional Asian diet, is linked to a lower obesity prevalence and obesity related pathologies [15].

The protective effects elicited by genistein could be related to its effect on adipogenesis, which would be inhibited by mechanisms related to 5’ adenosine monophosphate-activated protein kinase α/β (AMPKα/β), [1,16,17]. Also, the anti-inflammatory and antioxidant properties exerted by genistein in different cell types [18,19,20] could represent a mechanism leading to its protective effects. So far however, information about those issue is scarce. Moreover, no data is available about its effects on brown adipocytes.

Thus, in the present study we examined the effects of genistein on human visceral pre-adipocytes differentiation towards white and brown adipose tissue, as well as its actions on cell viability, mitochondrial membrane potential and oxidative stress of pre-adipocytes and white and brown adipocytes. Moreover, the role of extracellular signal regulated phospho-Akt, mitofusin-2 (Mfn2), and AMPKα/β expression or activation was analyzed.

## 2. Materials and Methods

### 2.1. Isolation of Pre-Adipocyte from Adipose Tissue

Human VAT samples were collected from 30 subjects undergoing general surgery for diverticulitis and laparocele. Ethical approval and informed consent were obtained prior to surgery (The study was approved by the Ethics Committee of the University Hospital Maggiore of Charity of Novara. All participants provided written informed consent prior to participation). Men aged 50–70 were included. Exclusion criteria were as follows: inability to give informed consent, underlying malignancy, endocrinological disorders, diabetes, metabolic syndrome, smoking, and intake of cardiovascular drugs, statins, hormonal agents, and corticosteroids. VAT tissue, approximately 10–20 g, was the excess adipose tissue surrounding surgical specimens that have been removed during surgical procedures. Human pre-adipocytes were isolated from adipose tissue by first washing the tissue sample extensively with phosphate-buffered saline (PBS) containing 5% penicillin/streptomycin (P/S; Sigma, Milan, Italy). In order to extract pre-adipocytes, the sample was minced using two scalpels and pipetted up and down several times to facilitate the digestion in a sterile tissue culture plate with 2 mg/mL collagenase Type I (Life Technologies, Monza, Italy) prepared in PBS containing 2% P/S for tissue digestion and then put at 37 °C with 5% CO_2_ in incubator. After 2 h, collagenase Type I activity was neutralized by adding to the tissue sample an equal volume of pre-adipocyte medium alpha minimum essential medium (α-MEM; Sigma) supplemented with 20% fetal bovine serum (FBS; Euroclone, Pero, Milan, Italy), 2 mM L-glutamine (Sigma) and 1% penicillin-streptomycin (Sigma). The sample was then transferred to a 50 mL tube, avoiding the solid aggregates and centrifuged at 2000 rpm for 5 min. After spinning, all the collagenase solution above the pellet was aspirated and resuspended in 3 mL complete α-MEM supplemented with 20% FBS, and the cell suspension was filtered through 70 mm cell strainer. The sample containing the cells was plated and incubated at 37 °C, 5% CO_2_ [21].

### 2.2. Culture, Expansion and Treatment of Pre-Adipocytes

Seventy-two hours after plating, the medium was aspirated from the plate. The cells were washed with PBS supplemented with 1% P/S. The solution over the cell layer was pipetted several times in order to clean the cells thoroughly from any tissue fragments and/or blood cells. A volume of fresh α-MEM 10% FBS was added according to the well capacity of the culture plate. The cells were maintained at 37 °C with 5% CO_2_. The medium was changed every second day until the cells reached 80–90% confluence and then pre-adipocytes were used to perform the experiments.

For mitochondrial membrane potential measurement and cell viability, 20 × 10^4^ cells/well were plated in 12-well plates with complete α-MEM 10% FBS. The same procedure was followed for reactive oxygen species (ROS) quantification, but using 30 × 10^3^ cells/well. For Oil-Red O Staining Kit (BioVision, Inc., San Francisco, CA, USA), 10 × 10^4^ cells/well were plated in 24-well plates. For Western Blot, 40 × 10^4^ cells/well were used in 6-well plates. A pool of cells at confluence, which was considered as day 0, was induced to differentiate by using α-MEM medium supplemented with hormone cocktail containing 0.5 mM 3-isobutyl-1 methylxanthine (IBMX; Sigma), 0.5 µM dexamethasone (Sigma), 50 µM indomethacin (Santa Cruz Biotechnology, Inc., Santa Cruz, CA, USA). This medium was taken as differentiation medium (DM) as previously performed [9,21]. After 48 h, this medium was replaced every 2 days. Droplets began to appear 8 days after inducing differentiation and by day 14 cells containing large lipid droplets were considered mature adipocytes [9]. In addition, another pool of cells was also treated with 1 nM 3,3′,5-Triiodo-l-thyronine sodium salt (T3; Sigma), which was added to DM in order to induce brown differentiation [6,9]. Following the procedure described above, cells were maintained in this medium until exhibiting fully differentiated phenotype with massive accumulation of multilocular fat droplets. All experiments were performed within 15 passages.

### 2.3. Oil-Red O Staining

We examined anti-obesity potential of genistein by determining pre-adipocyte differentiation into adipocytes. Pre-adipocytes were treated with either genistein (10 pM, 1 µM, 50 µM and 200 µM; Sigma) or adiponectin (100 µM; Sigma) for all the differentiating period. Control cells were treated with DM only. On day 14, differentiation was over and fat drops were detected by Oil-Red O staining (BioVision). According to the manufacturer’s instructions, the cells were washed with PBS and fixed in 10% formalin for 30 min. After washing twice with PBS and once with 60% isopropanol, the cells were then stained with Oil Red O Working Solution and incubated for 20 min. Afterward, the cells were washed with dH_2_O until the excess stain was no longer evident and stained for nuclei with hematoxylin solution for 1 min. At the end, cells were washed with dH_2_O and viewed under the microscope. After staining with hematoxylin and washing with dH_2_O, cells were washed three times with 60% isopropanol. Oil Red O stain was extracted with 100% isopropanol and 80% of extraction volume was transferred in a 96-well to measure the absorbance at 492 nm by a spectrometer (BS1000 Spectra Count, San Jose, CA, USA). Experiments were repeated five times in pre-adipocytes taken from each patient.

### 2.4. Cell Viability

Cell viability was examined by using the 1% 3-[4,5-dimethylthiazol-2-yl]-2,5-diphenyl tetrazolium bromide (MTT; Life Technologies, Italia, Monza, Italy) dye, as previously described [19,22,23] both in pre-adipocytes and in white adipocytes (treated with DM) and in brown adipocytes (treated with DM supplemented with 1 nM T3). Pre-adipocytes/adipocytes were treated in physiological and peroxidative conditions with adiponectin (100 µM; Sigma) and genistein at different doses (10 pM, 1 µM, 50 µM and 200 µM; Sigma) for 30 min. Control cells were treated with α-MEM/DM only. Oxidative stress was generated in pre-adipocytes/adipocytes by 200 μM hydrogen peroxide (H_2_O_2_) for 30 min alone or in the presence of genistein and adiponectin. The antioxidant N acetyl-cysteine (NAC; Sigma) was used as positive control. In some experiments the effects of 200 μM genistein on cell viability were also examined in the presence of equimolar wortmannin and dorsomorphin (Sigma), the Akt and AMPKα/β inhibitors, respectively. After each treatment, the treatment medium was removed, and fresh culture medium α-MEM without FBS and with MTT dye was added in 24-well plates containing the cells and incubated for 2 h at 37 °C in an incubator. Thereafter, the medium was removed, and 50 µL dimethyl sulfoxide (DMSO; Sigma) was added and mixed in a gyratory shaker until the complete dissolution of formazan crystals. Cell viability was determined by measuring the absorbance through a spectrometer (BS1000 Spectra Count), and cell viability was calculated by comparing results with control cells. Experiments were repeated five times in pre-adipocytes taken from each patient.

### 2.5. Mitochondrial Membrane Potential Measurement

Mitochondrial membrane potential measurement was performed with JC-1 assay as previously described for cell viability. After stimulation, the treatment medium was removed and cells were incubated with 5,51,6,61-tetrachloro-1,11,3,31 tetraethylbenzimidazolyl carbocyanine iodide (JC-1) 1X diluted in Assay Buffer 1X for 15 min at 37 °C, following the manufacturer’s instruction and as previously performed (Invitrogen, Life Technologies Europe BV, Monza, Italy) [19,22,23]. After incubation, the cells were washed twice with Assay Buffer 1× and then the mitochondrial membrane potential was determined by measuring the red (excitation 550 nm/emission 600 nm) and green (excitation 485 nm/emission 535 nm) fluorescence through a spectrometer (BS1000 Spectra Count). To assess cells that had apoptosis, the ratio of red to green fluorescence was determined and expressed as a percentage. Experiments were repeated five times in pre-adipocytes taken from each patient.

### 2.6. ROS Quantification

The oxidation of 2,7-dichlorodihydrofluorescein diacetate (H2DCFDA) into 2,7-dichlorodihydrofluorescein (DCF) was used to assess ROS generation, following the manufacturer’s instructions (Abcam, Cambridge, UK). Experimental protocol was the same as that followed for cell viability and mitochondrial membrane potential but without the use of inhibitors. After treatments, the reactions were stopped by removing the medium and washing with PBS, which was followed by staining with 10 µM H2DCFDA for 20 min at 37 °C. The fluorescence intensity of H2DCFDA was measured at an excitation and emission wavelength of 485 nm and 530 nm using a spectrometer, as previously performed (BS1000 Spectra Count) [19,22,23]. Experiments were repeated five times in pre-adipocytes taken from each patient.

### 2.7. Cell Lysates

For Western blot analysis, pre-adipocytes/adipocytes were treated in physiological and peroxidative conditions with genistein (10 pM and 200 µM; Sigma) and adiponectin (100 µM; Sigma) as described above. At the end of stimulation, pre-adipocytes/adipocytes were lysed in iced-Ripa-buffer supplemented with sodium orthovanadate (2 mM; Sigma) and protease inhibitors cocktail (1 mM; Thermo Fisher Scientific, Waltham, MA, USA) and phenylmethanesulfonyl fluoride (1 mM; Sigma). The extracted proteins were quantified through bicinchoninic acid protein (BCA, Pierce, Rockford, IL, USA) and used for electrophoresis and immunoblotting studies. Experiments were repeated five times in pre-adipocytes taken from each patient.

### 2.8. Western Blotting

Cell lysates (30 μg protein each sample) dissolved in Laemmli buffer 5×, boiled for 5 min were resolved in 10% sodium dodecyl sulfate polyacrylamide gel electrophoresis gels (SDS-PAGE, Bio-Rad Laboratories, Hercules, CA, USA). After electrophoresis they were transferred to polyvinylidene fluoride membranes (PVDF, Bio-Rad Laboratories), which were incubated overnight at 4 °C with specific primary antibodies: anti phospho-Akt (p-Akt; 1:1000; Ser473, Santa Cruz Biotechnology, Inc., Santa Cruz, CA, USA), anti Akt (1:1000; Santa Cruz Biotechnology), anti UCP1 (1:1000; 4E5, Santa Cruz Biotechnology), anti p-AMPKα/β (1:1000; D-6, Santa Cruz Biotechnology), AMPKα/β (1:1000; Santa Cruz Biotechnology), anti Mfn2 (1:1000; H-68, Santa Cruz Biotechnology). The membranes were washed and then incubated with horseradish peroxidase-coupled goat anti-rabbit IgG (Sigma), peroxidase-coupled rabbit anti-goat IgG and horseradish peroxidase-coupled goat anti-mouse IgG (Sigma) for 45 min and were developed through a nonradioactive method using Western Lightning Chemiluminescence (PerkinElmer Life and Analytical Sciences, Waltham, MA, USA). Phosphorylated protein expression was calculated as a ratio towards specific total protein or β-actin (1:5000; C4, Santa Cruz Biotechnology).

### 2.9. Statistical Analysis

All data were recorded using the Institution’s database. Statistical analysis was performed by using STATVIEW version 5.0.1 for Microsoft Windows (SAS Institute Inc., Cary, NC, USA). Data were checked for normality before statistical analysis. All the results obtained were examined through one-way ANOVA followed by Bonferroni post hoc tests. The non-parametric Mann Whitney U test for unpaired data was used to compare percentage responses. All data are presented as means ± standard deviation (SD) of five independent experiments for each experimental protocol executed in adipocytes taken from each patient. A value of *p* < 0.05 was considered statistically significant.

## 3. Results

### 3.1. Effects of Genistein on Human Visceral Pre-Adipocytes Differentiation in Physiological Conditions

**Figure 1:**
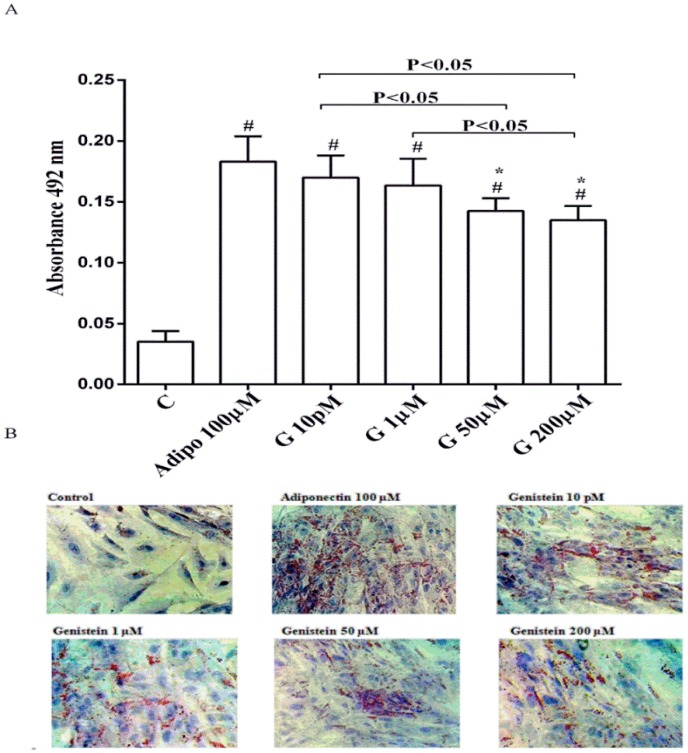
Quantitative (**A**) and qualitative (**B**) analysis of the effects of genistein on pre-adipocytes differentiation by using Oil Red O Staining: In A, effects of genistein (G) 10 pM, 1 µM, 50 µM and 200 µM, adiponectin (Adipo; 100 µM) for all the differentiating period. C = control (pre-adipocytes treated only with differentiation medium). Reported data are means ± SD of five independent experiments for each experimental protocol executed in pre-adipocytes taken from each patient. In A, ^#^ *p* < 0.05 vs. control; * *p* < 0.05 vs. Adipo 100 µM. Short square brackets indicate significance between groups (*p* < 0.05). In (**B**), in each panel one example for each treatment is reported. The images were taken at 10 × magnification.

As shown in Figure 1, in physiological conditions 10 pM and 1 µM genistein increased human visceral pre-adipocytes differentiation in a similar way to adiponectin. In contrast, the differentiation observed with genistein 50 µM and 200 µM was lower, although still higher than the control.

### 3.2. Effects of Genistein on Cell Viability and Mitochondrial Membrane Potential in Human Visceral Pre-Adipocytes/Adipocytes Cultured in Physiological and Peroxidative Conditions

**Figure 2:**
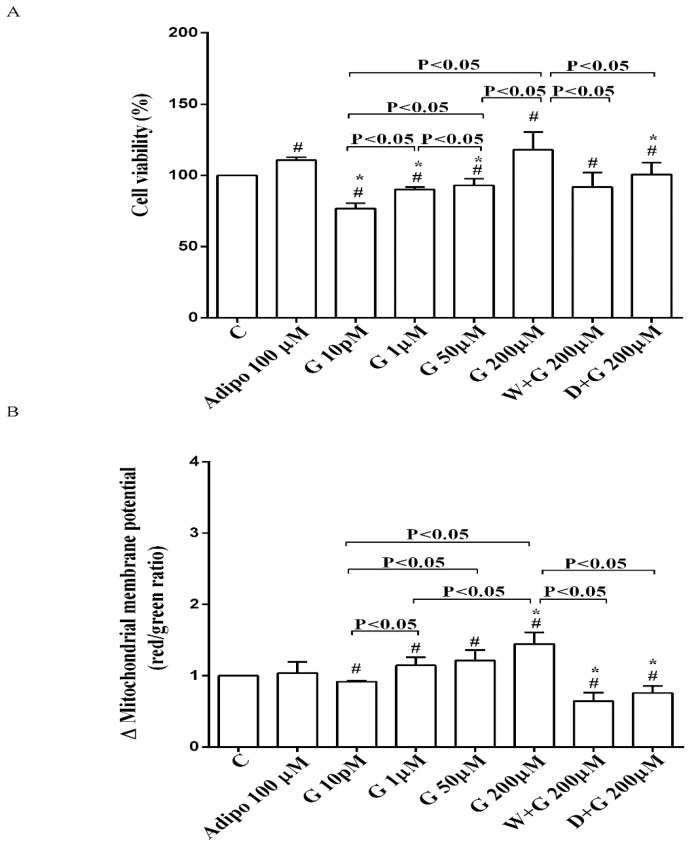
Effects of genistein in pre-adipocytes, cultured in physiological condition, on cell viability (**A**) and mitochondrial membrane potential (**B**): In A and B, the effects of genistein (G) 10 pM, 1 µM, 50 µM and 200 µM, adiponectin (Adipo; 100 µM), wortmannin (W; 200 µM), dorsomorphin (D; 200 µM). C = control. Reported data are means ± SD of five independent experiments for each experimental protocol executed in adipocytes taken from each patient. Significance between groups: ^#^ *p* < 0.05 vs. control; * *p* < 0.05 vs. Adipo 100 µM. Short square brackets indicate significance between groups (*p* < 0.05).

As shown in Figure 2A, in pre-adipocytes cultured in physiological conditions, adiponectin increased cell viability of about 11% in comparison with the control, without affecting mitochondrial membrane potential (Figure 2B). Genistein reduced cell viability up to 50 µM and increased it at 200 µM (Figure 2A). Moreover, an improvement of mitochondrial membrane potential was observed from 1 µM genistein concentration upwards (Figure 2B). The effects of genistein were abolished by both wortmannin and dorsomorphin (Figure 2A,B).

**Figure 3:**
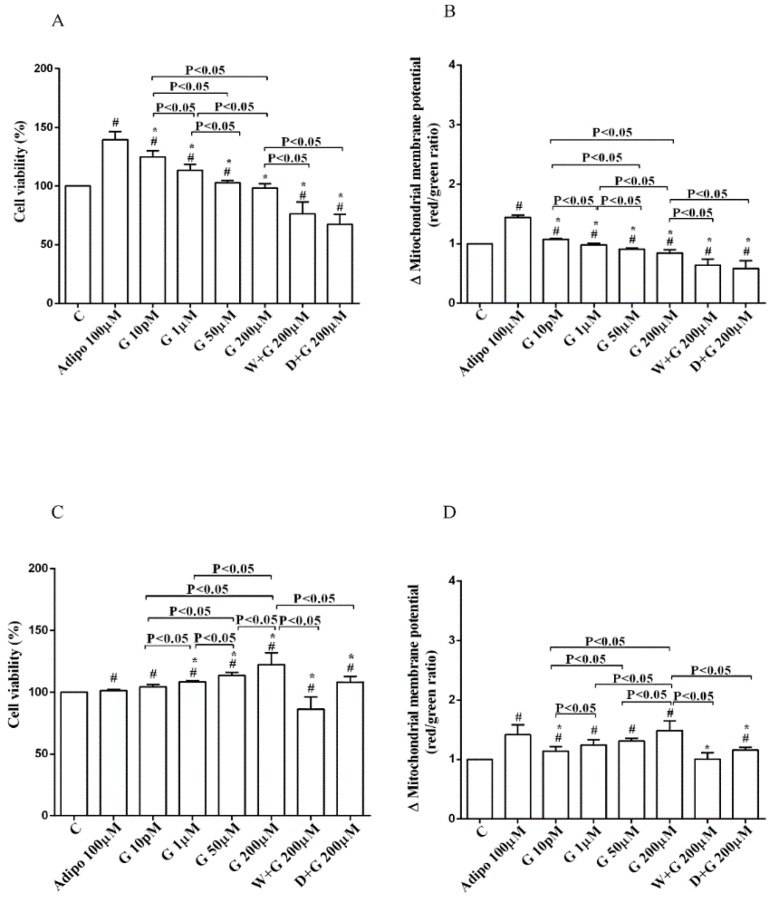
Effects of genistein in white adipocytes, cultured in physiological condition, on cell viability (**A**) and mitochondrial membrane potential (**B**), and in brown adipocytes, on cell viability (**C**) and mitochondrial membrane potential (**D**). Abbreviations are as in Figure 1 and Figure 2. Reported data are means ± SD of five independent experiments for each experimental protocol executed in adipocytes taken from each patient. Significance between groups: ^#^ *p* < 0.05 vs. control; * *p* < 0.05 vs. Adipo 100 µM. Short square brackets indicate significance between groups (*p* < 0.05).

Regarding white adipocytes cultured in physiological conditions, adiponectin augmented cell viability and mitochondrial membrane potential respectively by about 39% and 44% (Figure 3A,B). Also, genistein increased cell viability at concentration up to 50 µM (Figure 3A) in a way that was inversely related to the dose. However, only 10 pM genistein caused an improvement of mitochondrial membrane potential (Figure 3B). As observed for pre-adipocytes, wortmannin, and dorsomorphin abolished the increase of cell viability caused by genistein (Figure 3A).

Regarding brown adipocytes cultured in physiological conditions, a slight increase of cell viability and an improvement of about 41% in mitochondrial membrane potential were found in response to adiponectin. Also, genistein caused a concentration-dependent increase of both cell viability (Figure 3C) and mitochondrial membrane potential (Figure 3D). Those effects were abolished in brown adipocytes treated with wortmannin and dorsomorphin (Figure 3C,D).

**Figure 4:**
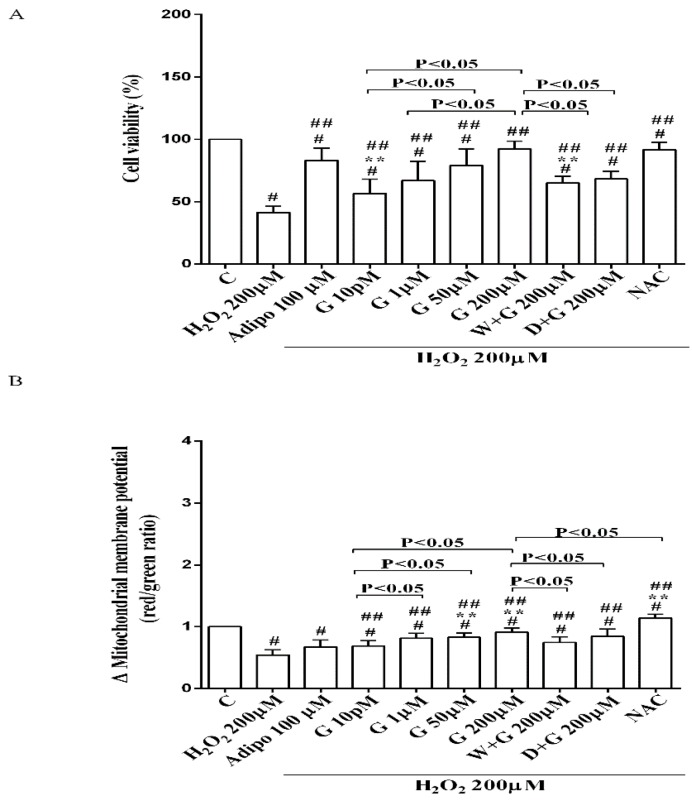
Effects of genistein in pre-adipocytes, cultured in peroxidative condition, on cell viability (**A**) and mitochondrial membrane potential (**B**). N acetyl-cysteine (NAC, 200 µM). Other abbreviations are as in previous figures. Reported data are means ± SD of five independent experiments for each experimental protocol executed in adipocytes taken from each patient. Significance between groups: ^#^ *p* < 0.05 vs. control; ^##^ *p* < 0.05 vs. H_2_O_2_ 200 µM; ** *p* < 0.05 vs. Adipo 100 µM + H_2_O_2_ 200 µM. Short square brackets indicate significance between groups (*p* < 0.05).

**Figure 5:**
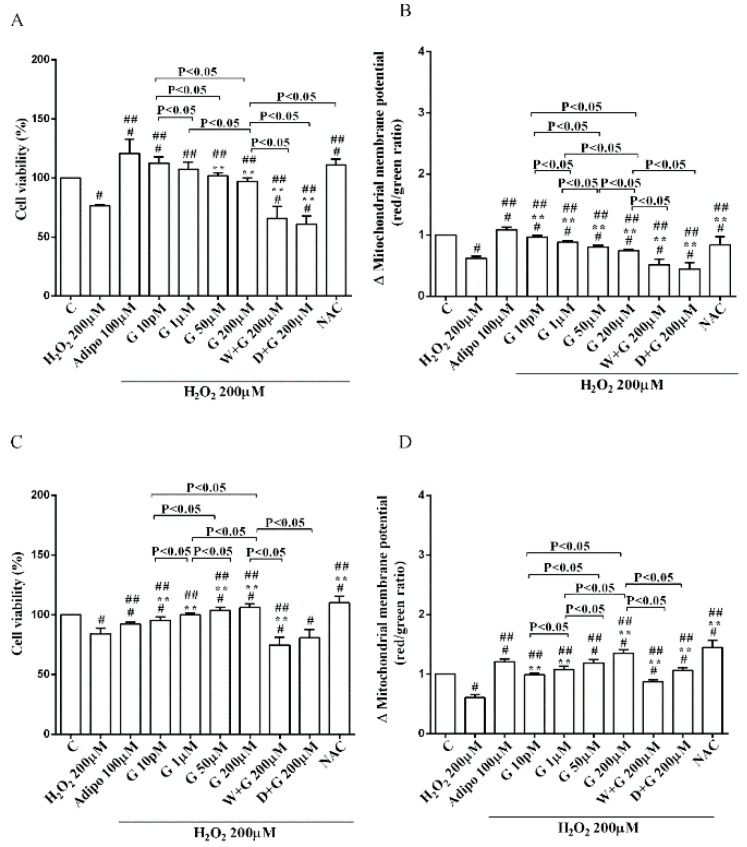
Effects of genistein in white adipocytes, cultured in peroxidative condition, on cell viability (**A**) and mitochondrial membrane potential (**B**), and in brown adipocytes, on cell viability (**C**) and mitochondrial membrane potential (**D**). Abbreviations are as in previous figures. Reported data are means ± SD of five independent experiments for each experimental protocol executed in adipocytes taken from each patient. Significance between groups: ^#^ *p* < 0.05 vs. control; ^##^ *p* < 0.05 vs. H_2_O_2_ 200 µM; ** *p* < 0.05 vs. Adipo 100 µM + H_2_O_2_ 200 µM. Short square brackets indicate significance between groups (*p* < 0.05).

Furthermore, in pre-adipocytes (Figure 4A), adiponectin counteracted the effects of 200 µM hydrogen peroxide on cell viability. In white and brown adipocytes, similar findings were observed not only on cell viability but also on mitochondrial membrane potential (Figure 5). As observed with adiponectin, also genistein exerted protection against peroxidation. In particular, in pre-adipocytes, all doses of genistein increased cell viability (Figure 4A) and mitochondrial membrane potential (Figure 4B). In white adipocytes, the effects on cell viability of genistein were similar to those of adiponectin for concentration up to 1 µM (Figure 5A). In respect of mitochondrial membrane potential, the effects of genistein were lower than the ones caused by adiponectin and were inversely related to the dose (Figure 5B). In brown adipocytes, all doses of genistein were more effective than adiponectin in increasing cell viability (Figure 5C). In contrast, only 200 µM genistein exerted higher effects on mitochondrial membrane potential in comparison with adiponectin (Figure 5D).

It is notable that the protective effects elicited by 200 μM genistein on cell viability were similar to those caused by 200 μM NAC in pre-adipocytes and brown adipocytes (Figure 4A and Figure 5C). Also regarding mitochondrial membrane potential, in white and brown adipocytes 200 μM genistein and 200 μM NAC elicited similar protective effects (Figure 5B,D). In contrast, in pre-adipocytes, NAC was more effective than genistein in preventing the collapse of mitochondrial membrane potential (Figure 4B).

### 3.3. Effects of Genistein on ROS Production in Human Visceral Pre-Adipocytes/Adipocytes Cultured in Peroxidative Conditions

**Figure 6:**
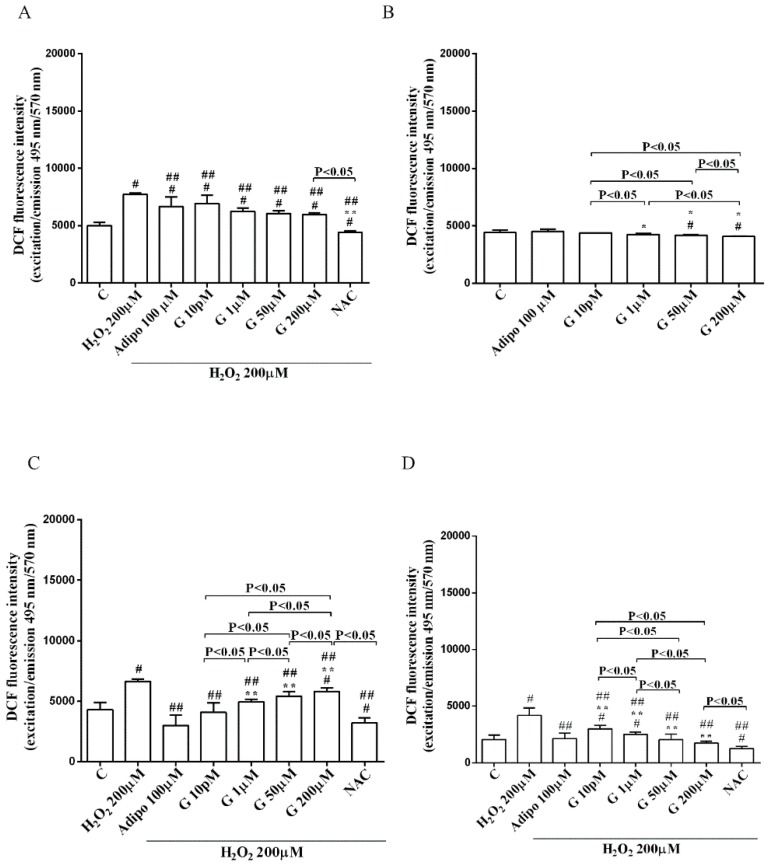
Effects of genistein on ROS production in pre-adipocytes, cultured in peroxidative (**A**) and physiological (**B**) conditions, and in white (**C**) and brown (**D**) adipocytes. Abbreviations are as in previous figures. Reported data are means ± SD of five independent experiments for each experimental protocol executed in adipocytes taken from each patient. Significance between groups: ^#^ *p* < 0.05 vs. control; * *p* < 0.05 vs. Adipo 100 µM; ^##^ *p* < 0.05 vs. H_2_O_2_ 200 µM; ** *p* < 0.05 vs. Adipo 100 µM + H_2_O_2_ 200 µM. Short square brackets indicate significance between groups (*p* < 0.05).

As shown in Figure 6, adiponectin reduced ROS production caused by 200 µM H_2_O_2_ in pre-adipocytes (Figure 6A) and in both white (Figure 6C) and brown (Figure 6D) adipocytes. Those effects were lower than those caused by NAC (Figure 6A,C,D). Moreover, in white adipocytes, genistein reduced ROS production caused by 200 µM H_2_O_2_ in a way that was inversely related to the dose. Similar findings were observed in pre-adipocytes and brown adipocytes although they were related to the dose of genistein. It is to note that in physiological conditions, only concentration higher than 50 µM genistein was able to reduce ROS release (Figure 6B).

### 3.4. Expression of UCP1 in Human Visceral Pre-Adipocytes/Adipocytes Cultured in Physiological and Peroxidative Conditions

**Figure 7:**
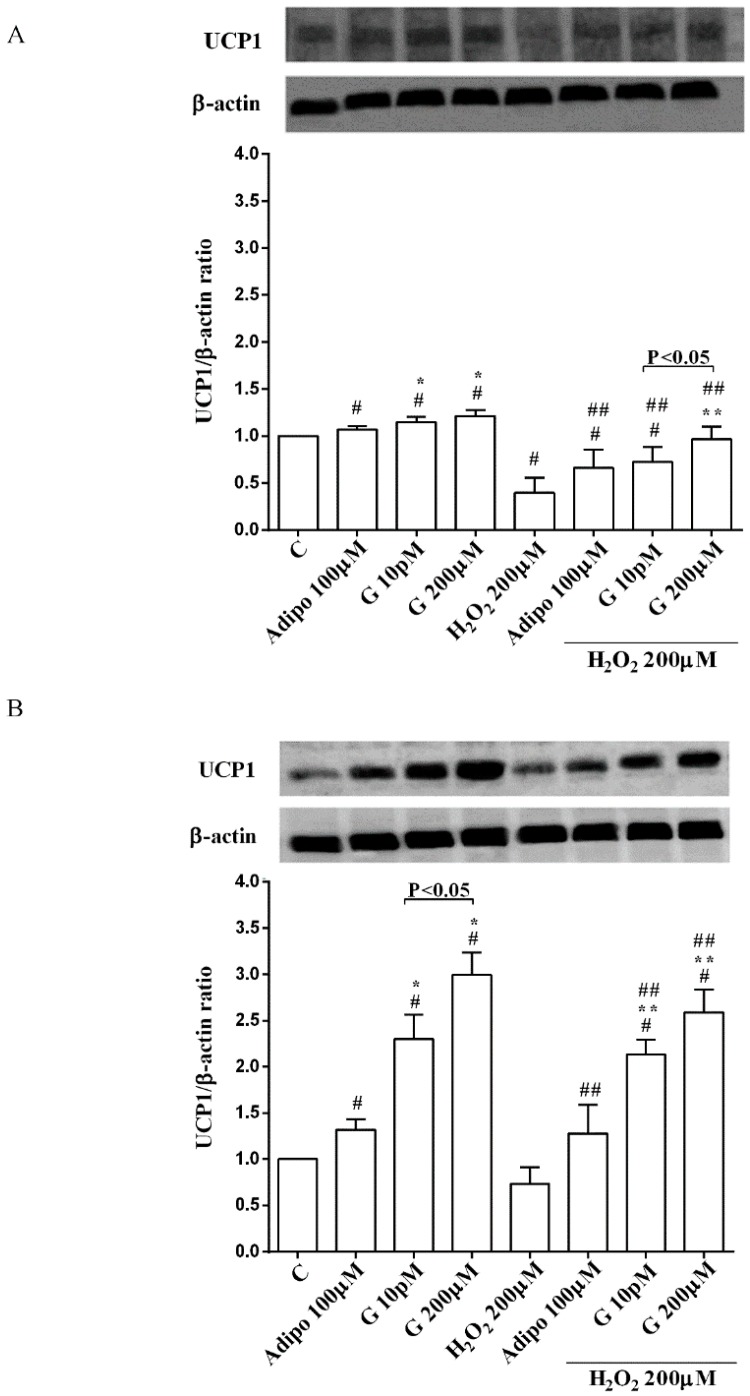
Effects of genistein on UCP1 activation in pre-adipocytes (**A**), and brown adipocytes (**B**), cultured in physiological and peroxidative conditions: Abbreviations are as in previous figures. Reported data are means ± SD of five independent experiments for each experimental protocol executed in adipocytes taken from each patient. Significance between groups: ^#^ *p* < 0.05 vs. control; * *p* < 0.05 vs. Adipo 100 µM; ^##^ *p* < 0.05 vs. H_2_O_2_ 200 µM; ** *p* < 0.05 vs. Adipo100 µM + H_2_O_2_ 200 µM. Short square brackets indicate significance between groups (*p* < 0.05).

Adiponectin and genistein increased UCP1 expression in pre-adipocytes and brown adipocytes cultured in physiological and peroxidative conditions (Figure 7A,B). The effects of genistein were higher than those caused by adiponectin in brown adipocytes only.

### 3.5. Involvement of Akt, Mfn2, and AMPKα/β in the Effects of Genistein in Human Visceral Pre-Adipocytes/Adipocytes Cultured in Physiological and Peroxidative Conditions

**Figure 8:**
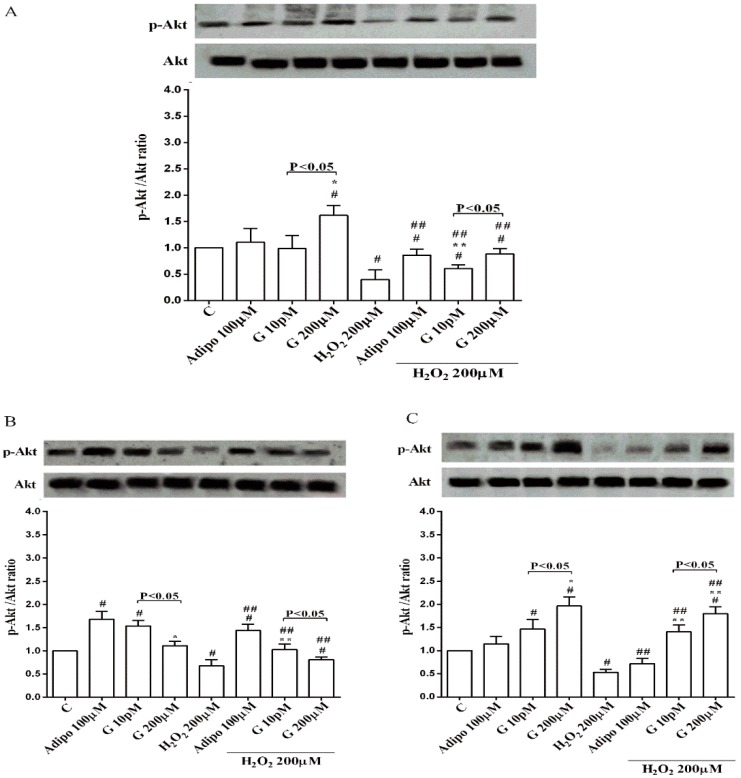
Effects of genistein on Akt activation in pre-adipocytes (**A**), white adipocytes (**B**) and brown adipocytes (**C**), cultured in physiological and peroxidative conditions: Abbreviations are as in previous figures. Reported data are means ± SD of five independent experiments for each experimental protocol executed in adipocytes taken from each patient. Significance between groups: ^#^ *p* < 0.05 vs. control; * *p* < 0.05 vs. Adipo 100 µM; ^##^ *p* < 0.05 vs. H_2_O_2_ 200 µM; ** *p* < 0.05 vs. Adipo 100 µM + H_2_O_2_ 200 µM. Short square brackets indicate significance between groups (*p* < 0.05).

**Figure 9:**
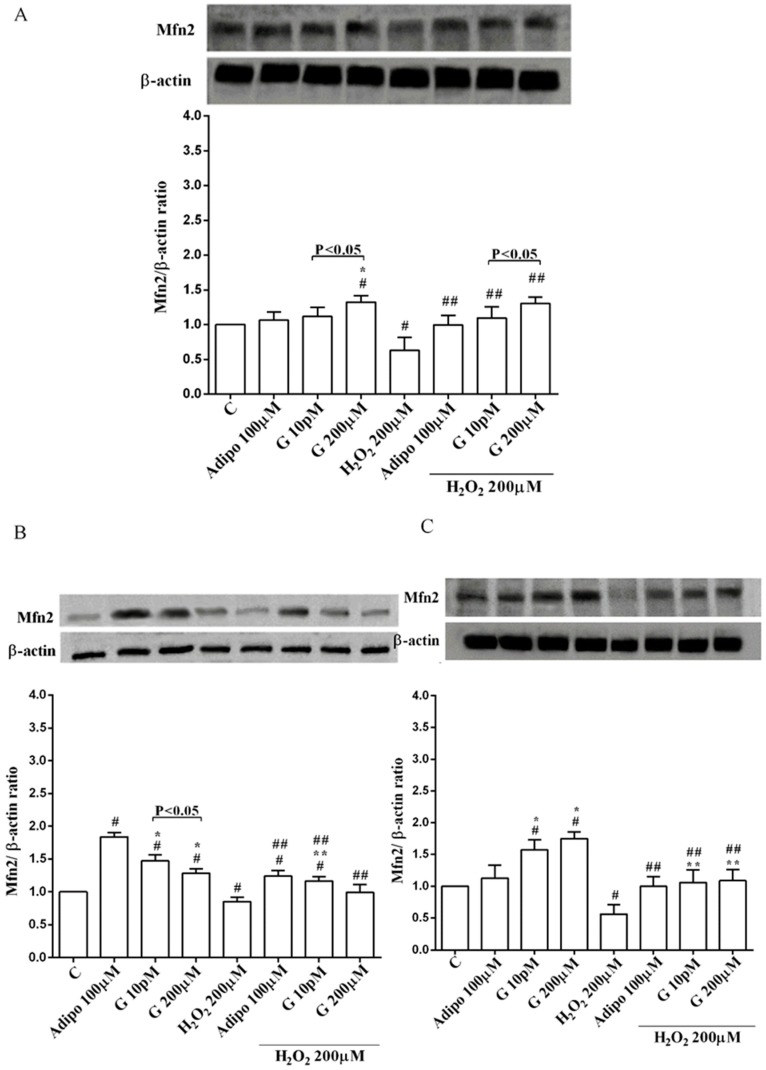
Effects of genistein on Mfn2 expression in pre-adipocytes (**A**), white adipocytes (**B**) and brown adipocytes (**C**), cultured in physiological and peroxidative conditions: Abbreviations are as in previous figures. Reported data are means ± SD of five independent experiments for each experimental protocol executed in adipocytes taken from each patient. Significance between groups: # *p* < 0.05 vs. control; * *p* < 0.05 vs. Adipo 100 µM; ## *p* < 0.05 vs. H2O2 200 µM; ** *p* < 0.05 vs. Adipo 100 µM + H2O2 200 µM. Short square brackets indicate significance between groups (*p* < 0.05).

**Figure 10:**
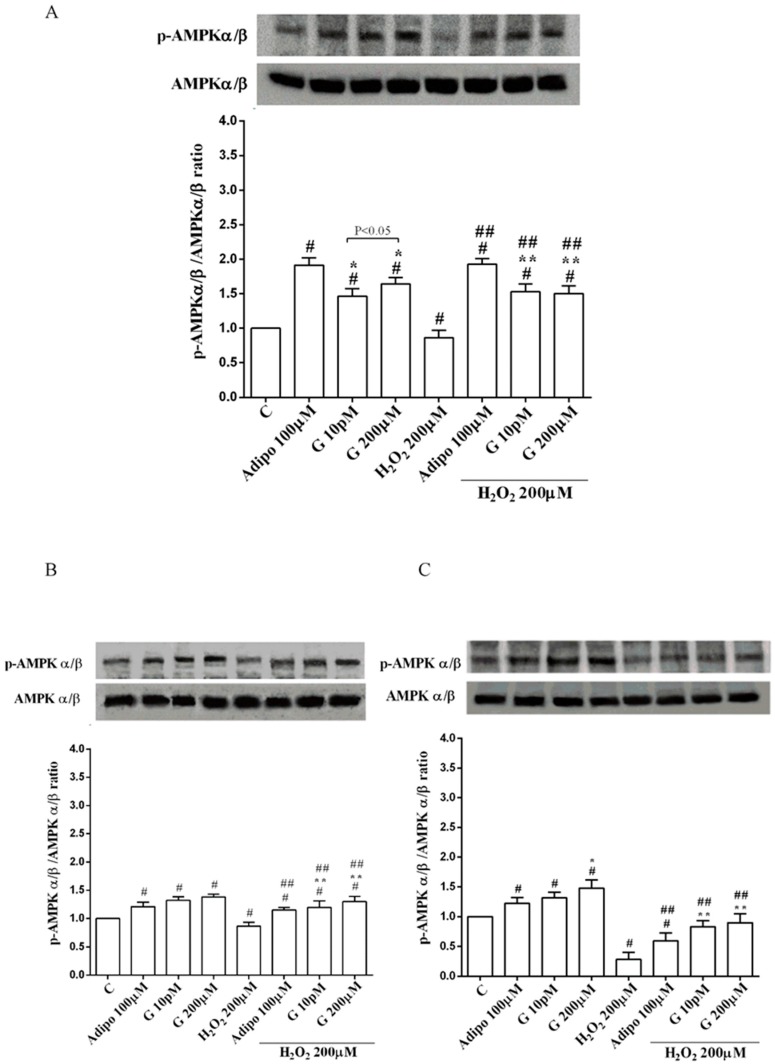
Effects of genistein on AMPKα/β activation in pre-adipocytes (**A**), white adipocytes (**B**) and brown adipocytes (**C**), cultured in physiological and peroxidative conditions: Abbreviations are as in previous figures. Reported data are means ± SD of five independent experiments for each experimental protocol executed in adipocytes taken from each patient. Significance between groups: # *p* < 0.05 vs. control; * *p* < 0.05 vs. Adipo 100 µM; ## *p* < 0.05 vs. H2O2 200 µM; ** *p* < 0.05 vs. Adipo 100 µM + H2O2 200 µM. Short square brackets indicate significance between groups (*p* < 0.05).

Adiponectin increased Akt activation in white (Figure 8B) and brown (Figure 8C) adipocytes, and Mfn2 expression in white adipocytes only (Figure 9B). In pre-adipocytes, cultured in physiological conditions, only 200 µM genistein was able to increase Akt activation and Mfn2 expression (Figure 8A and Figure 9A). In addition, while in white adipocytes cultured in physiological conditions only 10 pM genistein augmented Akt activation (Figure 8B), all doses of genistein exerted this effect in brown adipocytes (Figure 8C). It is also to note that in adipocytes, an increase of Mfn2 expression was observed in response to all doses of genistein (Figure 9B,C). Finally, both adiponectin and genistein prevented the effects of hydrogen peroxide on Akt activation and Mfn2 expression (Figure 8 and Figure 9). On the other hand, in pre-adipocytes (Figure 10A), and in white (Figure 10B) and in brown (Figure 10C) adipocytes, the pretreatment with genistein and adiponectin improved the activation of AMPKα/β and counteracted the effects of H_2_O_2_.

## 4. Discussion

This study has shown that genistein increases differentiation and browning of human visceral pre-adipocytes and exerts positive effects on cell viability and mitochondrial membrane potential in either white or brown adipocytes. Furthermore, genistein is effective in protection against peroxidation by inhibition of ROS release and preserving mitochondrial function, as shown by Mfn2 expression modulation in both pre-adipocytes and adipocytes.

Genistein, the most abundant soy-derived isoflavone that shares structural similarities with 17 β estradiol has various biological activities, amongst which is the inhibition of adipogenesis in various cellular models [14,24,25,26]. Its inhibitory effects on adipocyte metabolism and maturation could be the underlying mechanism of its protection against cardiovascular diseases or metabolic syndrome [27].

The results we have obtained in human visceral pre-adipocytes are in disagreement with previous observations, since the pretreatment of cells with genistein at doses that have been widely used for similar studies [28] increased pre-adipocytes differentiation, as shown by Oil Red staining. Those effects were not different from the ones elicited by adiponectin, for genistein concentration up to 1 μM. Meanwhile, genistein increased the browning of pre-adipocytes, as shown by the augmented UCP1 expression [29,30].

Also, our results regarding Mfn2 and mitochondrial membrane potential in human visceral pre-adipocytes treated with genistein could be considered as a marker of browning. Our data would be in agreement with previous studies, showing that Mfn2 is highly expressed in BAT, where it would act as a mediator of mitochondrial interaction with lipid droplets and as a modulator of whole-body energy homeostasis [31,32].

The discovery that white adipocytes can undergo a browning process to become metabolically active beige cells has attracted significant interest in the fight against obesity [33]. Hence, differently from white adipocytes, which are responsible for energy storage, brown adipocytes are key regulators in the dissipation of chemical energy as heat. In this process, UCP1, a widely accepted genetic marker of brown adipocytes, [29] would be highly involved [30].

As previously shown, AMPKα/β was found to be activated by genistein in human visceral pre-adipocytes. AMPKα/β represents a metabolite-sensing protein kinase [34] that is widely involved in the regulation of energy homeostasis in various ATP-depleting metabolic states, such as those found in hypoxia and oxidative stress [35]. For this reason, AMPKα/β cascades have emerged as novel targets for the treatment of obesity and type 2 diabetes [36]. It is to note that AMPKα/β activation was reported to be implicated in the inhibition of adipocyte differentiation [37] by mechanisms related to increased ROS release [1].

In our study, genistein augmented AMPKα/β activation in human visceral pre-adipocytes cultured in physiological condition while causing no effect or reducing ROS generation, which could account for the differences we have found in differentiation. A more complex role played by AMPKα/β in the modulation of adipogenesis through ROS release would be involved. It is also of note that our results were obtained in human primary cells and not in 3T3-L1 pre-adipocytes, as performed in those studies. Thus, differences in the biological behavior related to the cellular type and different experimental conditions could account for the discrepancies we have found.

Moreover, since Akt activation would play an important role in human adipogenesis [38] our results about Akt could at least explain the effects of the highest dose genistein on human pre-adipocytes differentiation, too.

It is of note that, unlike genistein, the effects of adiponectin on human visceral pre-adipocytes differentiation were only accompanied by an increase AMPKα/β activation and of UCP1 expression. The finding about increased adipogenesis of adiponectin is in agreement with the previous ones obtained in 3T3-L1 [39]. Also, the effects of adiponectin on AMPKα/β would confirm previous data, although they were related to the lipolytic action exerted by the adipokine in adipose tissue [40].

Further studies would thus be necessary to clarify the cross talk between AMPKα/β and Akt in adipogenesis in human visceral adipose tissue.

In adipocytes, different results were obtained between white or brown cells. Hence, in white adipocytes cultured in physiological conditions an inverse dose related response on cell viability was observed. Only a slight increase in mitochondrial membrane potential were found with 10 pM genistein. In brown adipocytes, the protective effects on cell viability were related to the dose and all doses genistein improved mitochondrial membrane potential. Those effects were accompanied by an increased activation of Akt and by an augmented expression of Mfn2, which was particularly evident for brown adipocytes, as mentioned above. In peroxidative conditions, genistein protected both white and brown adipocytes against the lost cell viability and the collapse of mitochondrial membrane potential. This protection was also evident in human visceral pre-adipocytes. Moreover, a reduction of hydrogen peroxide-related ROS release was found. It is to note that although genistein was less effective than NAC in reducing ROS release, it was able to exert similar protective effects on cell viability and mitochondrial membrane potential as those elicited by NAC, particularly in brown adipocytes, which would highlight the protective role of genistein as antioxidant agent.

As observed for pre-adipocytes, also in adipocytes the effects elicited by genistein were partly similar to those caused by adiponectin. Hence, in white and brown adipocytes the adipokine increased cell viability, mitochondrial membrane potential and augmented Akt and AMPKα/β activation; however only an increase of Mfn2 was found in white adipocyte. The involvement of Akt and AMPKα/β was confirmed by experiments performed with specific inhibitors. Hence, in the presence of wortmannin and dorsomorphin the effects of genistein were generally reduced or abolished in either pre-adipocytes or white/brown adipocytes. Moreover, a protection against peroxidation was observed. Our data add new information about those issues. Hence, few reports available in chicken adipocytes have shown that adiponectin could prevent the reduction of lipid-induced mitochondrial biogenesis by AMPK related signaling [41]. AMPK would also be involved in its effects on the insulin sensitivity of 3T3-L1 adipocytes. [42].

In addition, the data obtained with JC-1 would be in agreement with previous findings showing that the preservation of mitochondrial function could play a role in the protection exerted by adiponectin [43].

Regarding the mechanisms of action leading to the beneficial effects exerted by genistein, a role for Akt, AMPKα/β and Mfn2 could be hypothesized. Indeed, Akt is a well-known member of the reperfusion injury salvage kinase pathway that could play an essential role in the modulation of insulin sensitivity of adipocytes. In 3T3-L1 adipocytes, paraquat-induced oxidative stress decreased glucose transporter 4 translocation to the cell surface by inhibition of the phosphorylation of Akt [44]. Although not clearly examined, and in agreement with previous findings [45], our data showing the increased phosphorylation of Akt would highlight a beneficial role for genistein in preserving insulin signaling and glucose uptake against oxidative stress in human adipocytes, as well. Also, in the case of adiponectin, the protective effects observed against peroxidation in white and brown adipocytes could be related to the increased Akt activation. These data would, on the other hand, confirm previous ones about the beneficial effects exerted by adiponectin on the cardiovascular system [46,47].

As mentioned above, Mfn2 is a mitochondrial membrane protein that is crucial in mitochondrial fusion, and loss of its function can greatly damage the mitochondrial network in several cell types. It is also to note that Mfn2 has recently been described as a regulator of hormone-stimulated lipolysis by acting as an A-kinase anchoring protein on the surface of the lipid droplets [48]. Therefore, changes in Mfn2 observed in oxidative stress conditions could account for the altered lipid metabolism found in the metabolic syndrome or diabetes [49]. Our data would be in agreement with those observations. Hence, in our study, genistein counteracted the reduction of Mfn2 expression caused by peroxidation in human visceral pre-adipocytes and adipocytes. Those effects were accompanied by the preservation of a mitochondrial function and reduced ROS release. Further experiments would be necessary to better understand the role of Mfn2 in mediating the beneficial effects of genistein in lipid metabolism in human visceral adipocytes.

All together our findings would highlight a role for genistein as a promising compound for the treatment of diabetes or metabolic disorders through the modulation of mitochondrial network. Hence, genistein would increase the browning in human visceral pre-adipocytes and exert protection against oxidative damage in pre-adipocytes and mature adipocytes too, by mechanisms related to Akt and AMPKα/β activation and the modulation of mitochondrial fusion.
